# Supplementary material for: A T-Cell-Derived 3-Gene Signature Distinguishes SARS-CoV-2 from Common Respiratory Viruses
Source: Viruses. 2024 Jun 26;16(7):1029. doi: 10.3390/v16071029 (PMC11281602; doi:10.3390/v16071029)
Supplement: Supplementary file 1 [file viruses-16-01029-s001.zip › Supplementary Materials.docx]

A T-Cell-Derived 3-Gene Signature Distinguishes SARS-CoV-2 from Common Respiratory Viruses

LI et al.,

**Supplementary method**

## Identification of SARS-CoV-2 specific 3-gene signature via integrative bioinformatics and machine learning approaches

To develop SARS-CoV-2 specific signature with high accuracy and stability performance, we integrated five machine learning algorithms with the ability of feature selection, including random forest, XGBoost, generalized boosted regression modeling (GBM), least absolute shrinkage and selection operator (LASSO) and elastic network. The random forest, XGBoost and GBM were applied in python library sciki-learn (v1.2.1), xgboost (v1.7.3) and lightgbm (v3.3.5). The LASSO and elastic network were performed in R package “glmnet” (v4.1-4).

The random forest model had two parameters *n_estimators* and *max_features* where *n_estimators* represented the number of trees in the forest and *max_features* was the number of features to consider when looking for the best split. The XGBoost model had two parameters *learning_rate* and *n_estimators* where *learning_rate* was the the step size shrinkage used to prevent overfitting and *n_estimators* shared similar functions in random forest. The GBM model had two similar parameters in XGBoost, *learning_rate* and *n_estimators*. We used a grid-search on these parameters using 10-fold cross-validation. For LASSO and elastic network models, the regularization parameter, lambda, was determined by 10-fold cross-validation, whereas the L1-L2 trade-off parameter, α, was set to 0-1 (interval =0.1).

Figure S1. Evaluation of data Integration with HRA000786 and GSE17156. The principal component analysis (PCA) before (A) and after (B) batch effect correction on HRA000786 (SARS-CoV-2 infections) and GSE17156 (HRV, IFV and RSV independent challenge cohort).

Figure S2. Scale-free fit index and mean connectivity described for various soft-thresholding powers.


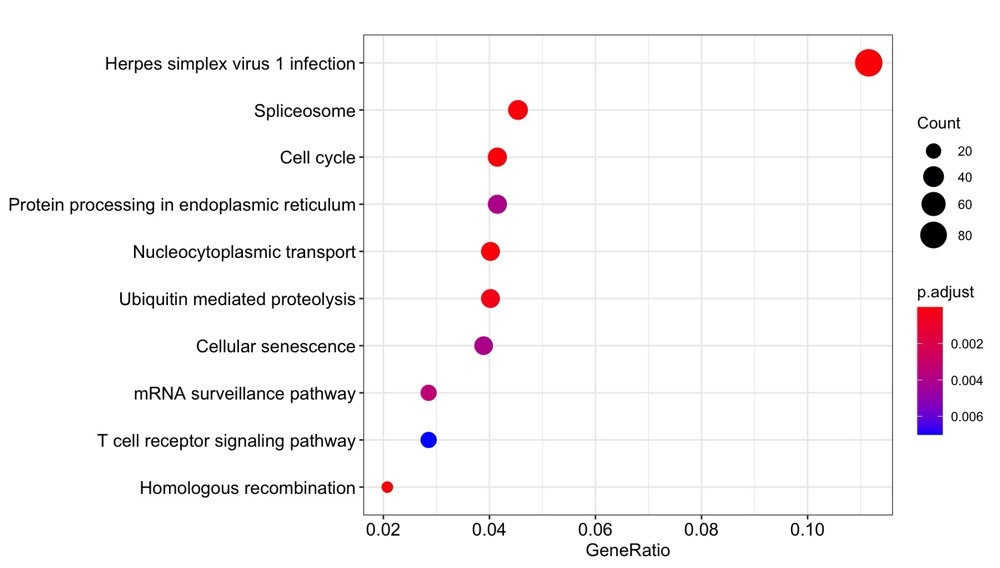


Figure S3. The most enriched KEGG terms in blue module. KEGG: Kyoto Encyclopedia of Genes and Genomes.

Figure S4. Scatter plot of eigengenes from the candidate genes of SARS-CoV-2 infection and other viral infections.


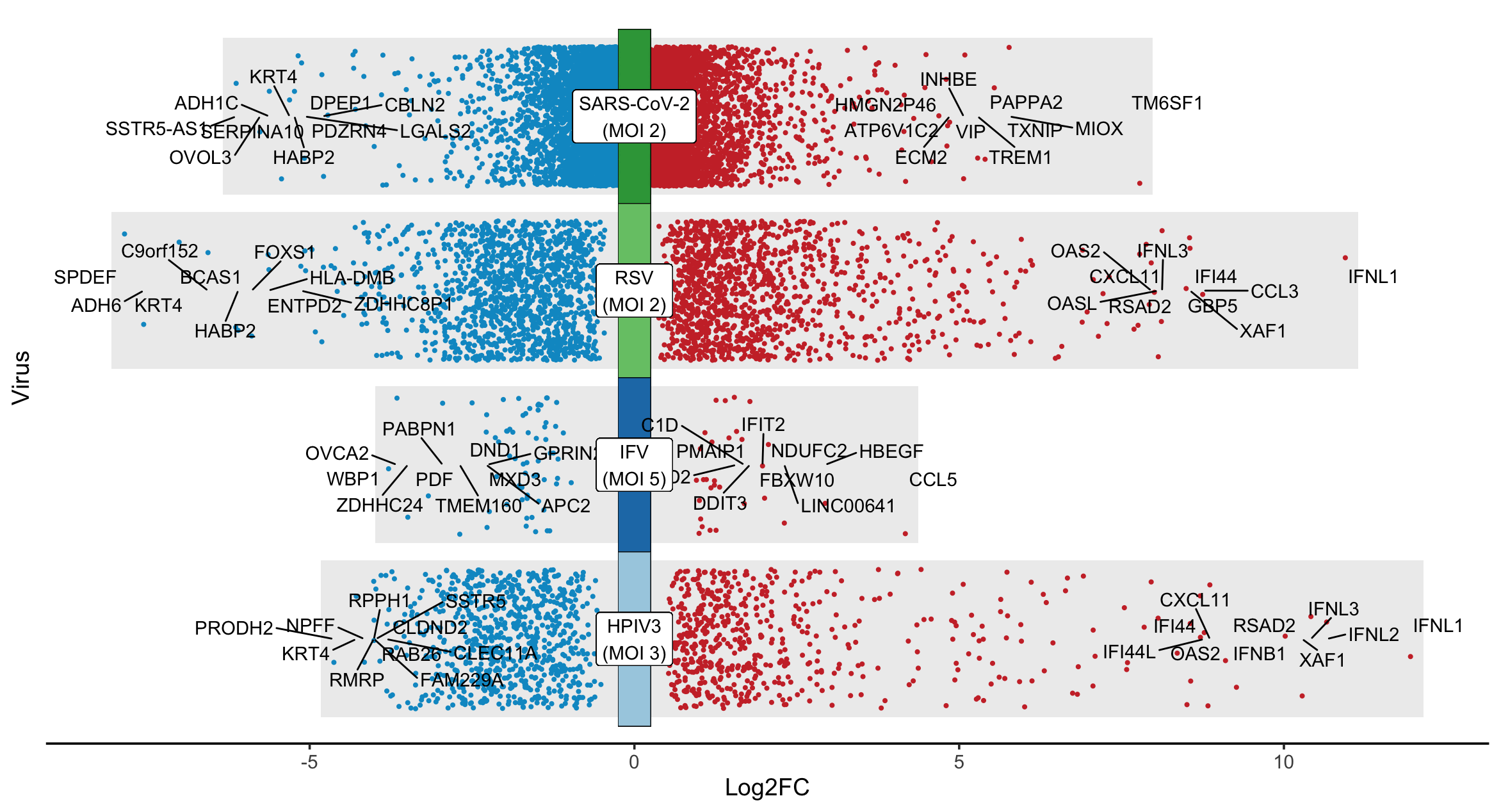


Figure S5. Differential expression genes (DEGs) of A549 cells with viral infections at high MOI. Data were retrieved from GSE147507. MOI, multiplicity of infection; HPIV3, human parainfluenza virus 3.
